# Drug Target Commons: A Community Effort to Build a Consensus Knowledge Base for Drug-Target Interactions

**DOI:** 10.1016/j.chembiol.2017.11.009

**Published:** 2018-02-15

**Authors:** Jing Tang, Zia-ur-Rehman Tanoli, Balaguru Ravikumar, Zaid Alam, Anni Rebane, Markus Vähä-Koskela, Gopal Peddinti, Arjan J. van Adrichem, Janica Wakkinen, Alok Jaiswal, Ella Karjalainen, Prson Gautam, Liye He, Elina Parri, Suleiman Khan, Abhishekh Gupta, Mehreen Ali, Laxman Yetukuri, Anna-Lena Gustavsson, Brinton Seashore-Ludlow, Anne Hersey, Andrew R. Leach, John P. Overington, Gretchen Repasky, Krister Wennerberg, Tero Aittokallio

**Affiliations:** 1 Institute for Molecular Medicine Finland (FIMM), University of Helsinki, 00014 Helsinki, Finland; 2 Department of Mathematics and Statistics, University of Turku, 20014 Turku, Finland; 3 Chemical Biology Consortium Sweden, Karolinska Institutet, 17165 Stockholm, Sweden; 4 European Molecular Biology Laboratory, European Bioinformatics Institute (EMBL-EBI), Hinxton CB10 1SD, UK; 5 Medicines Discovery Catapult, Mereside, Alderley Park, Alderley Edge, Cheshire SK10 4TG, UK

**Keywords:** drug discovery, drug repurposing, chemical biology, cheminformatics, crowd sourcing, data curation, bioassay annotation, drug repositioning, community effort, open data

## Abstract

Knowledge of the full target space of bioactive substances, approved and investigational drugs as well as chemical probes, provides important insights into therapeutic potential and possible adverse effects. The existing compound-target bioactivity data resources are often incomparable due to non-standardized and heterogeneous assay types and variability in endpoint measurements. To extract higher value from the existing and future compound target-profiling data, we implemented an open-data web platform, named Drug Target Commons (DTC), which features tools for crowd-sourced compound-target bioactivity data annotation, standardization, curation, and intra-resource integration. We demonstrate the unique value of DTC with several examples related to both drug discovery and drug repurposing applications and invite researchers to join this community effort to increase the reuse and extension of compound bioactivity data.

## Highlights

• DTC is a crowd-sourcing-based web platform to annotate drug-target bioactivity data
• The open environment improves data harmonization for drug repurposing applications
• DTC offers a comprehensive, reproducible, and sustainable bioactivity knowledge base

## Graphical Abstract

**Figure:**
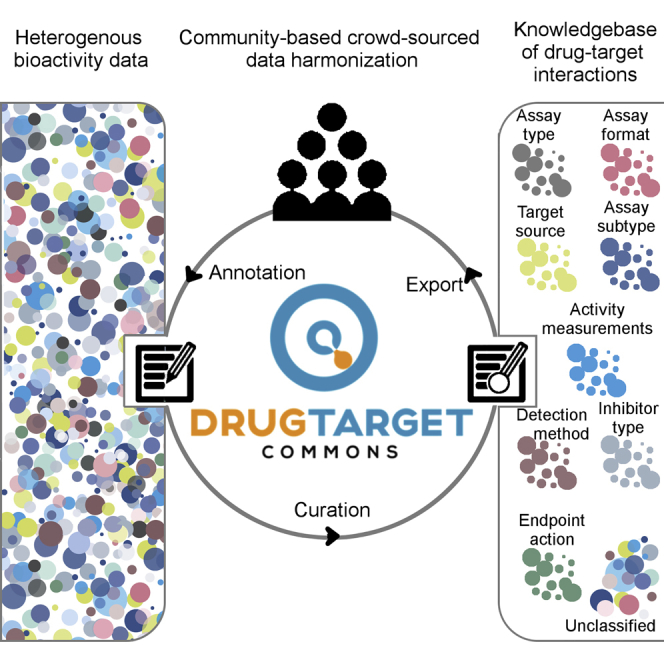

## Teaser

Tang et al. launches a novel crowd-sourcing effort to standardize the collection, management, curation, and annotation of the notoriously heterogeneous compound-target bioactivity measurements. The web-based community platform aims to provide the most comprehensive, reproducible, and sustainable bioactivity knowledge base for the end users.

## Introduction

Mapping the full spectrum of potential interactions between compounds and their targets, including both intended or “primary targets” as well as unintended secondary or “off-targets”, is a critical part of most drug discovery and development efforts, not only enabling exploration of the therapeutic potential of these chemical agents but also better understanding and management of their possible adverse reactions prior to clinical testing. Accumulating data show that most drugs bind to more than one target molecule within a biologically relevant affinity range (Santos et al., 2017). For instance, most kinase inhibitors bind to the conserved ATP-binding pocket of several or many distinct protein kinase domains that share sequence and structural similarity, leading to target promiscuity and a broad range of polypharmacological effects (Davis et al., 2011). Systems-wide compound-target interaction networks are therefore necessary to fully understand the mode of action of such promiscuous compounds, as well as to extend therapeutic uses of both approved and abandoned drugs, i.e., drug repurposing (Pemovska et al., 2015).

Efforts have been made to collect and curate quantitative compound-target interaction data, covering both active and inactive endpoints from various high- and low-throughput target-profiling experiments (Hersey et al., 2015, Santos et al., 2017, Wang et al., 2017). The diversity of specific profiling bioassays and approaches in drug discovery often leads to a high degree of data heterogeneity, commonly arising from the use of different experimental assays and bioactivity endpoints, as well as from differing detection technologies and endpoint measurements. Such non-standardized and heterogeneous experimental factors pose challenges for the comparison and integration of these bioactivity data resources, especially when using them to interpret and mine phenotypic profiling data for drug discovery and drug repurposing applications, an area of research that has gained significant traction in recent years (Arrowsmith et al., 2015, Schreiber et al., 2015, Plowright et al., 2017, Licciardello et al., 2017, Corsello et al., 2017).

**Figure 1:**
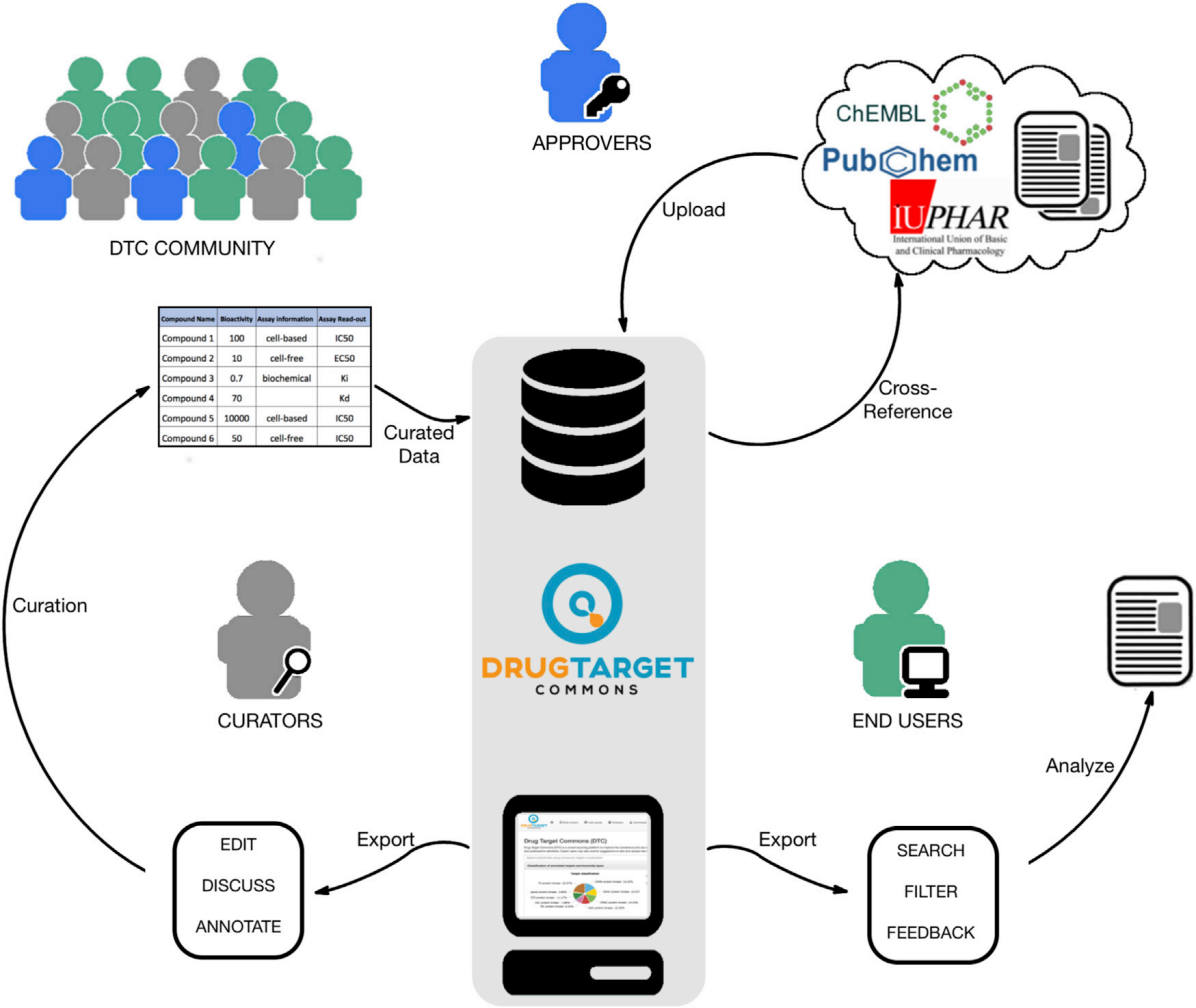
Schematics of the DTC Platform (Open-Access Database and Web Application) The web-based platform enables the user community to take part in crowd-sourced data extraction, annotation, and curation, as well as in using and analyzing comprehensive and standardized compound-target interaction profiles. The community-driven effort aims to provide maximally high-quality and reproducible bioactivity profiles and related information that will be fed back and cross-referenced to the original data sources, therefore supplementing and enhancing the coverage and annotation of existing drug/target data resources through the crowd-sourcing initiative. Processing errors and inconsistencies in the experimental data can be minimized via open discussions, enabled by the web interface, and only the most reliable bioactivity data will be released for end users through regular updates under the Creative Commons License.

To address these challenges, we implemented a web-based, open-access platform, called Drug Target Commons (DTC, https://drugtargetcommons.fimm.fi/), to initiate a community-driven crowd-sourcing effort to collectively extract, integrate, annotate, and standardize quantitative compound-target bioactivity data from the literature and other database sources (Figure 1). DTC implements a number of unique features including: (1) an interactive web interface enabling end users to not only upload new data from experiments or literature but also to participate in the data annotation and curation, together with the committed data approvers; (2) a specifically adapted compound-target bioactivity assay annotation and data curation procedure to provide more informative target profiles, making it possible to sort out inconsistencies between profiling studies that use differing assay types and endpoints; and (3) high-quality and comprehensive target profiles, which include not only the primary and secondary targets but also disease- or drug-response-related mutant targets, hence capturing the whole spectrum of potential target potencies. Compared with the existing data resources, the open-data environment and crowd-sourcing curation ensures that the most up-to-date experimental data for compound-target interactions will be sufficiently annotated and cross-checked before being approved and deposited into the DTC database (Table S1).

### Bioassay Annotation Explains Part of the Heterogeneity in Bioactivity Data

Differing experimental assays contribute to heterogeneous and irreproducible bioactivity data. For example, biochemical assays typically generate higher potency numbers than cell-based assays, especially for compounds that compete for binding with natural ligands or enzyme cofactors, such as ATP-competitive kinase inhibitors. However, large differences between biochemical and cellular potencies may also suggest that the compound does not penetrate the cell membrane, that it has undesirable protein-binding activities, or that it is not metabolically stable in cellular environments. To facilitate the standardization of bioassay annotation, we implemented a simplified version of the bioassay ontology (BAO) (Abeyruwan et al., 2014), termed μBAO (micro bioassay ontology), which conforms to the MIABE (minimum information about a bioactive entity) guidelines (Orchard et al., 2011) for describing compound-target bioactivities (Table S2). Compared with the original BAO, the simplified μBAO annotation allows the data curators to extract the assay information relatively quickly from the method descriptions of published literature (Figures S1 and S2).

As a proof of concept, we performed three rounds of data extraction and annotation processing with the DTC platform. These test rounds have already produced an extensive and standardized open-data resource that spans a broad spectrum of compound and target classes (Figures S3 and S4; Table S3). We initiated the μBAO annotation process based on the bioactivity assays that have already been deposited in ChEMBL, currently the most comprehensive, public-domain, and manually curated bioactivity database (Gaulton et al., 2017). These initial test rounds have resulted so far in 187,600 fully annotated bioactivity data points among 4,082 chemical compounds and across 528 distinct protein targets, with specific focus on kinase targets.

**Figure 2:**
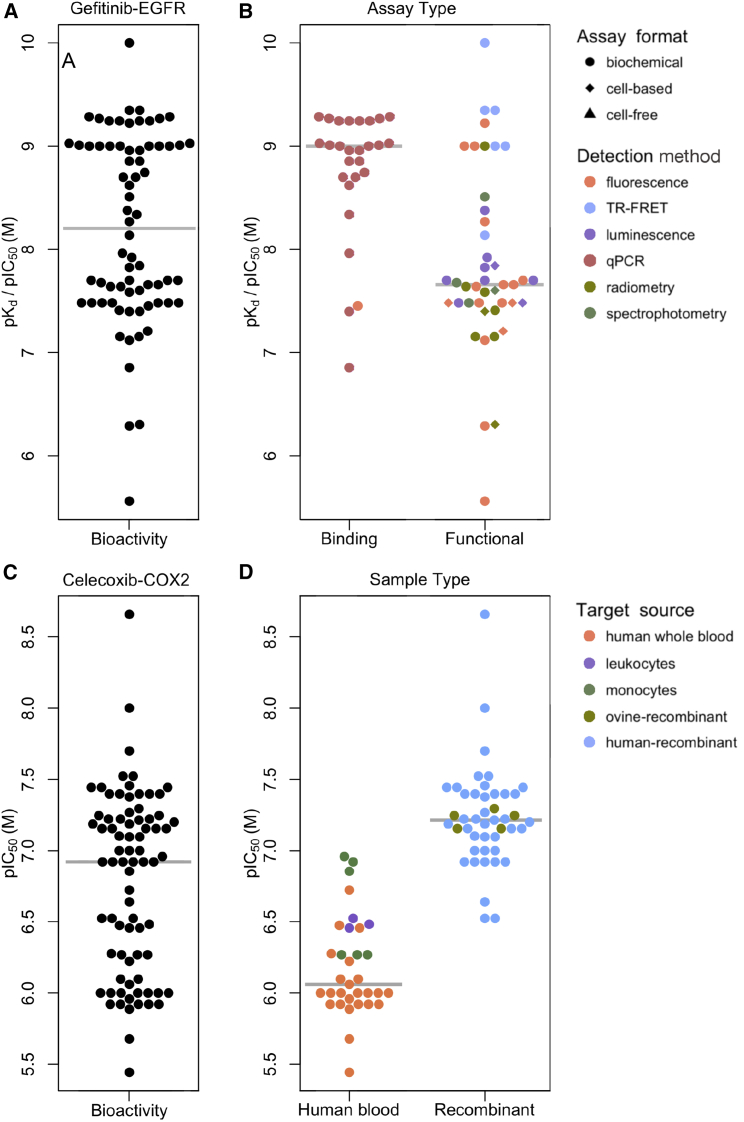
Bioassay Annotations Explain Heterogeneity in Bioactivity Data (A) 74 bioactivity data points for the gefitinib-EGFR drug-target pair prior to assay annotation. (B) The μBAO annotation process revealed that the major source of the variation was driven by the assay type (x axis), and further variation can be attributed to the detection technique and assay formats (colors and shapes). The low potency outliers originated from kinase assays run at very high ATP concentrations. (C) 78 bioactivity data points for the celecoxib-COX2 drug-target pair before assay annotations. (D) A clear distinction was observed in the assays performed *ex vivo* (human blood), compared with recombinant proteins (x axis). Further variation in the bioactivities arises from the specific target sources. See also Figures S1 and S2.

As a first specific example, we focused on gefitinib, an epidermal growth factor receptor (EGFR) tyrosine kinase inhibitor approved for treatment of patients with non-small-cell lung cancer. The available, nominally comparable gefitinib/EGFR interaction affinity data points (which in the initial release of DTC largely come from the data in ChEMBL) were spread out over a ∼10,000-fold concentration range (Figure 2A). However, the μBAO annotation enabled a better understanding of the heterogeneity in the gefitinib/EGFR bioactivity values (Figure 2B; see Figure S2 for examples). Specifically, the observed variability in the bioactivity data points was due primarily to functional assays that used different assay formats and detection technologies, whereas the binding assays showed more consistent potency values (Figure 2B). The extreme outliers in the functional assays originated from various detection methods. Of note, publication or deposition errors are beyond the scope of any assay annotation and may explain a portion of the remaining outlier data points (Kruger and Overington, 2012).

As a second case example, we carefully annotated a new set of bioactivities reported between the non-steroidal anti-inflammatory drug, celecoxib, and its primary target cyclooxygenase 2 (COX-2; gene symbol *PTGS2*). In doing so, we observed that adding information about the source of the target protein is essential for explaining the data heterogeneity (Figure 2C). Notably, assays using purified recombinant protein exhibited more than 10-fold higher potency than those measuring enzymatic activity in cellular extracts (Figure 2D). One explanation for the differing readouts may be that the high protein-binding propensity of celecoxib reduces its free concentration in the more heterogeneous cell-extract assays, and therefore results in a perceived lower target potency (Paulson et al., 1999), but there may also be other factors such as metabolic stability that contribute to this difference. This example emphasizes the importance of deep enough assay annotation for interpreting the heterogeneous bioactivity profiles.

### DTC Provides a High-Quality Knowledge Base to Facilitate Drug Repositioning

Among the 4,082 compounds we have annotated so far, we found interesting selectivity patterns that may help identify drug repurposing opportunities. Despite the high number of bioactivity data for some well-studied compounds, such as dasatinib, bosutinib, and staurosporine, each with more than 1,000 unique bioactivity records (Figure S5), the size of the potential target space is much smaller, with an average one potent target per compound (STAR Methods). When searching for potent inhibitors against given proteins, FLT3, AURKB, KDR, and FLT4 appear as the top-studied kinase targets, each having more than 120 active compounds (Figure S6A). Among mutated kinases, variants of BRAF and ABL1 emerge in the top tier, being targeted by more than 100 active compounds each (Figure S6B). Having access to both mutant and wild-type bioactivities enables mining compounds with a selective activity against a particular disease- or resistance-related kinase mutation.

**Figure 3:**
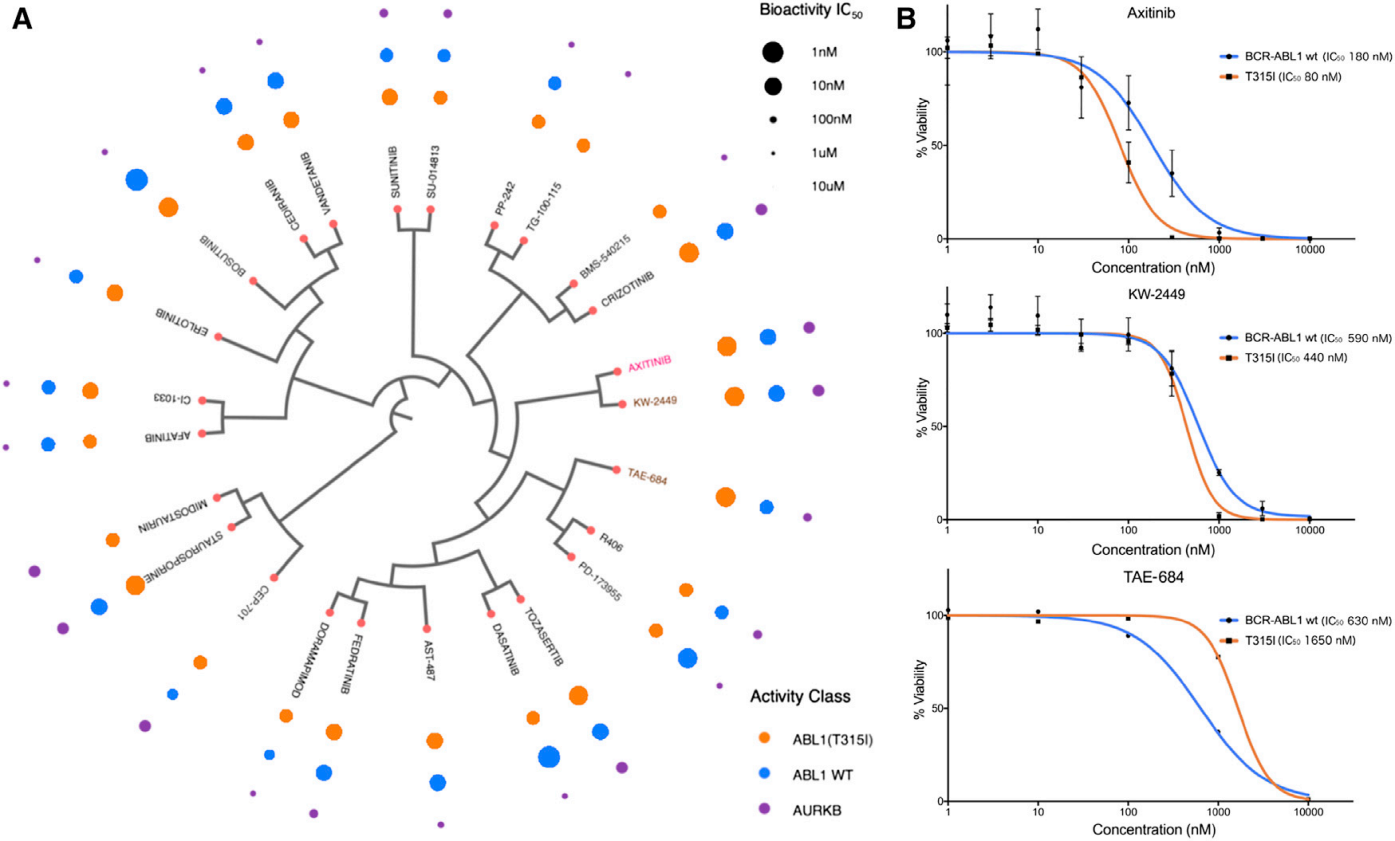
Compounds with Differential Potency against ABL1 (T315I) (A) A set of 25 compounds that showed potency toward phosphorylated-ABL1 (T315I), based on the current DTC database. Bubbles mark the potency class (based on half maximal inhibitory concentration [IC_50_] in nM) of these compounds toward ABL1 (T315I), wild-type ABL1, and Aurora kinase B (AURKB), as an estimate of the potential therapeutic window. The structural similarity of the compounds is visualized as a dendrogram (constructed with the C-SPADE web tool available at http://cspade.fimm.fi; Ravikumar et al., 2017). The gray-shaded part marks candidate compounds, KW-2449 and to a lesser extent TAE-684, that are structurally similar to axitinib (an ABL1 [T315I] inhibitor), and show similar differential selectivity toward ABL1 (T315I). (B) Ba/F3 cells stably expressing BCR-ABL1 (T315I) were used for experimental validation, with compound concentrations on the x axis and the viability readout on the y axis (mean and SD errors calculated based on three or more replicates). As expected, the positive control axitinib had a higher potency toward BCR-ABL1 (T315I)-driven cells, compared with BCR-ABL1 wild-type-driven cells; similarly, KW-2449 showed a slightly higher potency toward ABL1 (T315I) compared with BCR-ABL1 wild-type. The potency of TAE-684 was actually higher toward BCR-ABL1 wild-type than toward ABL1 (T315I) in the cell-based validation, demonstrating the importance of further pre-clinical evaluations before entering the drug optimization or repurposing phases.

As a case example, we focused on BCR-ABL1 fusion gene, given its importance in precision medicine for chronic myeloid leukemia (CML). Specifically, we took all the compounds in DTC that have reported potencies against ABL1 wild-type, BCR-ABL1(T315I), and Aurora B kinase, since inhibition of aurora kinases has turned out to be a dose-limiting toxicity-inducing off-target effect of BCR-ABL1(T315I) inhibitors (Goldenson and Crispino, 2015), and clustered them based on their structural similarities (Figure 3A). Such a target-specific tree provided enhanced information about the mutation-selective activities across a wide panel of approved and investigational compounds. For example, a VEGFR inhibitor axitinib was recently identified as a potent and selective BCR-ABL(T315I) inhibitor (Pemovska et al., 2015), and it is currently undergoing a clinical trial for CML (NCT02782403). Notably, compounds structurally similar to axitinib, including TAE-684 and KW-2449, also showed a strong potential to be repositioned as BCR-ABL1(T315I) inhibitors (highlighted in Figure 3A). Using a cell-based assay (Figure 3B), we confirmed that KW-2449, originally developed as an FLT3 inhibitor, is indeed active toward BCR-ABL1(T315I). However, we could not replicate the TAE-684 bioactivities, perhaps due to differing assay format compared with the original data source. This example shows how DTC data enable mapping of potential compound activities but also highlights the importance of cell-based target validation, ideally using multiple experimental assays, before entering into expensive and long drug development and clinical testing phases.

### An Invitation to Join the Collaborative Effort to Reuse and Extend Bioactivity Research Data

The major contributions of DTC as a drug discovery tool are the μBAO annotation and the crowd-sourcing web platform, which make it possible to utilize community power to enable deeper-level annotation of an extensive set of bioactivity assays, a process that is beyond the scope of any individual institute or group if working alone. The key to enable such a collaborative effort relies on effective communication and advertising, emphasizing the transparency, open-access, and ease-of-use of the DTC platform. The μBAO annotation system will be improved based on the emerging needs from the community, yet keeping it simple enough to allow for large-scale annotations. With an increasing number of data providers and curators joining this effort, we envision that the bioactivity data from public databases and newly published studies will be continuously annotated (Figure 1).

In the next phase, the fully annotated data will be cross-compared to reach a consensus view through community knowledge and evidence-based integration approaches (Knapp et al., 2013, Tang et al., 2014, Wang et al., 2016). We expect there will be a number of tools built on the DTC data by us and others that will provide added value from the bioactivity data, similar to the C-SPADE tool used in the present study for polypharmacological visualization (Ravikumar et al., 2017). In the long term, DTC will provide a sustainable open-access resource for many exciting applications, such as extending the space of the “druggable” cancer genome, not only for kinases but also for other target families including GPCRs, ion channels, and nuclear receptors. Comprehensive target selectivity profiles are also critical for the ongoing precision oncology efforts that use patient-specific mutation panels for tailoring targeted therapies (Dienstmann et al., 2015, Chakradhar, 2016, Griffith et al., 2017).

## Significance

**We have initiated Drug Target Commons (DTC) as a community-based crowd-sourcing platform designed to improve the reuse and consensus of compound-target bioactivity profiles. DTC implements an open environment to collectively curate, annotate, and integrate drug-target bioactivity data from literature and other resources. In this report, we demonstrated the added value and benefits of the DTC platform for application use cases relevant for drug discovery and repurposing applications. The deep-level expert curation and annotation as well as improved consensus on potency, selectivity, and therapeutic relevance of compounds are expected to greatly benefit many biological discovery, phenotypic profiling, and target deconvolution efforts in the future. To achieve the greatest impact, we invite chemical biologists, medicinal chemists, and computational biologists to join the community-driven data harmonization effort. As a return, all the curated data are freely available at** http://drugtargetcommons.fimm.fi**.**

## STAR★Methods

### Key Resources Table

**Table:** 

REAGENT or RESOURCE	SOURCE	IDENTIFIER
**Chemicals, Peptides, and Recombinant Proteins**		

Axitinib	LC Laboratories	Cat#A-1107
KW-2449	Selleck Chemicals	Cat#S2158
TAE684	MedChemExpress	Cat#HY-10192
Mouse IL-3 Recombinant Protein	eBioscience	Cat#14-8031-62

**Deposited Data**		

Bioactivity data and assay annotations	DTC website	https://drugtargetcommons.fimm.fi/

**Experimental Models: Cell Lines**		

Mouse: Ba/F3 parental cells (IL-3 dependent)	The Leibniz Institute DSMZ - German Collection of Microorganisms and Cell Cultures GmbH (DSMZ)	RRID:CVCL_0161
Mouse: Ba/F3 cells stably expressing BCR-ABL1	Tea Pemovska, (Pemovska et al., 2015)	NA
Mouse: Ba/F3 cells stably expressing BCR-ABL1 (T315I)	This paper	NA
Human: 90.74 (CRL-11654)	American Type Culture Collection (ATCC)	Cat#CRL-11654; RRID:CVCL_6361

**Recombinant DNA**		

pMIG-BCR-ABL1 plasmid	Prof. Dr. Nikolas von Bubnoff, University Medical Center Freiburg, Freiburg, Germany	NA
pMIG-BCR-ABL1 (T315I) plasmid	Prof. Dr. Nikolas von Bubnoff, University Medical Center Freiburg, Freiburg, Germany	NA

**Software and Algorithms**		

GraphPad Prism 7	GraphPad Prism Software, Inc.	https://www.graphpad.com/
Backend development technology: Python 3.4	DTC website	https://www.python.org/download/releases/3.4.0/
Frontend technology: Jquery 1.11.1, JavaScript	DTC website	https://blog.jquery.com/2014/05/01/jquery-1-11-1-and-2-1-1-released/
Postgresql 9	DTC database	https://www.postgresql.org/download/
Figures: MATLAB, R , Python	This paper	https://www.mathworks.com/products/new_products/release2016a.html https://www.rstudio.com/ https://www.python.org/download/releases/3.4.0/
C-SPADE	Figure 3 of this paper	http://cspade.fimm.fi
μBAO (micro bioassay ontology) protocol	This paper	NA

### Contact for Reagent and Resource Sharing

Further information and requests for resources and reagents should be directed to and will be fulfilled by the lead contact, Tero Aittokallio (tero.aittokallio@helsinki.fi)

### Experimental Model and Subject Details

Ba/F3 cells stably expressing BCR-ABL1 or BCR-ABL1 T315I were cultured in RPMI 1640 supplemented with 10% fetal bovine serum (FBS), L-glutamine and penicillin-streptomycin. For Ba/F3 parental cells (sex unknown), mouse recombinant interleukin-3 (IL-3; eBioscience) was added to the growth medium at a concentration of 10 ng/ml. The human virus-packaging cell line CRL-11654 (female) was cultured in Dulbecco's Modified Eagle's Medium (DMEM) containing 10% FBS and 1% penicillin-streptomycin. All cell lines were kept in 5% CO_2_ at 37°C. Ba/F3 parental cells (DSMZ) and CRL-11654 (ATCC) were purchased directly from their sources but were not authenticated.

### Method Details

#### Cell Line Assays

The Ba/F3 murine IL-3-dependent pro-B cell line was used to test kinase activity in a manner similar to previous studies (Warmuth et al., 2007). Ba/F3 cells stably expressing BCR-ABL1 were provided by Tea Pemovska and made as described here. Briefly, Ba/F3 cells stably expressing pMIG-BCR-ABL1 and pMIG-BCR-ABL1(T315I) plasmids made by infection with replication-incompetent retroviruses containing coding sequences for BCR-ABL1 and BCR-ABL1(T315I) collected after transfection of a virus-packaging cell line (CRL-11654, ATCC). 4 days post-infection, a stable expressing population was selected by removal of IL-3 for approximately 10 days. Ba/F3 cells stably expressing BCR-ABL1 or BCR-ABL1 (T315I) were then treated with a range of concentrations of axitinib, KW-2449 and TAE-684. Cell viability was detected by CellTiter-Glo Luminescent Cell Viability Assay (Promega) in a 384-well plate format. Luminescence was measured using a PHERAstar FS microplate reader (BMG Labtech), and half-maximal inhibitory concentration (IC_50_) was calculated (GraphPad Prism) to assess sensitivity of cell lines to the tested compounds. All assays were repeated at least three times, with consistent results.

##### Implementation Issues

The DTC platform was implemented to support a community-driven crowdsourcing effort to improve the consensus and use of biological target profiles of drugs and chemical tools (Figure 1). Common annotation terms are critical for standardizing biological experiments. To facilitate the data curation process, we implemented μBAO assay annotation that standardizes the description of target profiling experiments in terms of the assay type and format, endpoint type, detection technology, and other key determinants of the bioactivity readout (Figure S1). The web-based graphical user interface (GUI) enables end-users to search, view and download existing or community-annotated bioactivity data using a variety of compound, target or publication identifiers (see Data S1 for the user manual). Using the GUI, the expert users may also submit suggestions to edit or add new bioactivity data, as well as take part in the μBAO bioassay annotation and data curation process (see Data S2 for the glossary of annotation terms). Through the freely accessible DTC platform, the users cannot only upload new bioactivity data from their own experiments or literature, but can also participate in the process of data annotation, integration and quality-control, together with the committed domain experts. Such an open environment ensures that the experimental data points will be maximally curated, evaluated, and cross-checked before being deposited into the open DTC database for the downstream analyses.

#### Potent Bioactivities

We defined ’potent targets’ and ’potent inhibitors’ based on specific bioactivity cut-offs for the four most popular bioactivity types (K_d_, K_i_, IC_50_ and activity): ≤100 nM for the dose-response measurements (K_d_, K_i_ or IC_50_) in biochemical assays, and ≤1000 nM for the dose-response measurements (K_d_, K_i_ or IC_50_) in cell-based and other assay types. For the activity measurements (activity%, residual activity% or %inhibition), often resulting from assays with single or a few concentration points only, we defined a rather stringent threshold: ≤10% for the test concentration ≤1000 nM and ≤20% for test concentration of ≤500 nM in biochemical assays, and ≤50% for the test concentration ≤1000 nM and ≤10% for the test concentration ≤10000 nM in the cell-based assays. In cases where there were multiple bioactivity values for a compound-target pair, originating from different studies or other data resources, we took median bioactivity.

### Quantification and Statistical Analysis

No statistical analysis was performed on the cell line assay replicates.

### Data and Software Availability

All the bioactivity data points and annotations are freely available using application-programming interface (API) or direct download (CSV file) through DTC website: http://drugtargetcommons.fimm.fi (Download tab). See also https://drugtargetcommons.fimm.fi/userguide/ and https://drugtargetcommons.fimm.fi/glossary/. Details about source publications for fully annotated bioactivities are provided in Table S3.

## Author Contributions

J.T., K.W., and T.A. conceived the project and designed the study; Z.R.T. and G.P. developed the DTC database; Z.R.T. and Z.A. designed and implemented the web platform; Z.R.T managed data integration from various sources; J.T., Z.R.T., and G.R. coordinated the bioactivity data curation; Z.R.T., G.R., B.R., J.T., M.V.-K., A.J.A., J.W., A.J., E.P., E.K., P.G., L.H., G.P., L.Y., M.A., A.G., and S.K. performed the bioactivity data annotations. A.R. performed the cell line bioassays; A.H., A.R.L, J.P.O., A.-L.G. and B.S.-L. shared their in-house bioactivity data and helped with their annotation; B.R., Z.R.T., and J.T. implemented the data analyses; Z.R.T. and B.R. made the figures and tables for the manuscript; T.A. and J.T. drafted the manuscript; Z.R.T., B.R., A.R., G.R., K.W., A.H., A.R.L., and J.P.O. critically reviewed and edited the manuscript. All authors reviewed and approved the final version of the manuscript.

## Supplemental Information

Document S1. Figures S1–S6

Table S1. Comprehensive Listing of Related Drug/Target Data Resources and their Features, Related to Figure 1

Table S2. μBAO (Micro Bioassay Ontology) Terms as a Subset of BAO and Relation to MIABE, Related to Figure 2

Table S3. Main Sources of Bioactivity Data from Individual Studies in the Current DTC Release, Related to Figure 1

Data S1. User Manual for the DTC Web Interface, Related to STAR Methods

Data S2. Glossary of μBAO Annotation Terms, Related to STAR Methods

Document S2. Article plus Supplemental Information
