# Supplementary material for: Drug Target Commons: A Community Effort to Build a Consensus Knowledge Base for Drug-Target Interactions
Source: Cell Chem Biol. 2018 Feb 15;25(2):224–229.e2. doi: 10.1016/j.chembiol.2017.11.009 (PMC5814751; doi:10.1016/j.chembiol.2017.11.009)

Cell Chemical Biology, Volume *25*

Supplemental Information

Drug Target Commons: A Community Effort to Build a Consensus Knowledge Base for Drug-Target Interactions

Jing Tang, Zia-ur-Rehman Tanoli, Balaguru Ravikumar, Zaid Alam, Anni Rebane, Markus Vähä-Koskela, Gopal Peddinti, Arjan J. van Adrichem, Janica Wakkinen, Alok Jaiswal, Ella Karjalainen, Prson Gautam, Liye He, Elina Parri, Suleiman Khan, Abhishekh Gupta, Mehreen Ali, Laxman Yetukuri, Anna-Lena Gustavsson, Brinton Seashore-Ludlow, Anne Hersey, Andrew R. Leach, John P. Overington, Gretchen Repasky, Krister Wennerberg, and Tero Aittokallio

Supplementary Figures

Figure S1. μBAO assay annotation terminology. Related to Figure 1 and Data S2.

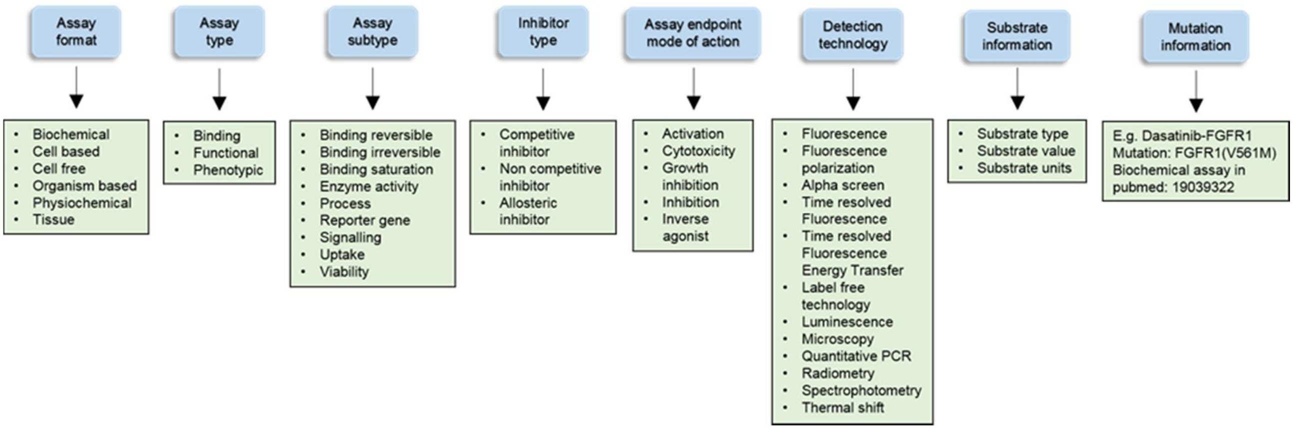

Figure S2. μBAO annotation examples for the gefitinib-EGFR bioactivity. Related to Figure 2.

Annotation of gefitinib (ChEMBL939)- EGFR drug-target pair		
	Example 1	Example 2
Compound ID	ChEMBL939	ChEMBL939
Target ID	P00533	P00533
Target preferred name	Epidermal growth factor receptor (erbB1)	Epidermal growth factor receptor (erbB1)
Standard binding type	IC50	Kd
Standard value	33	0.94
Standard units	nM	nM
Mutated/Wildtype	Wild type	Mutated target
Mutation information		EGFR(L834R)
Assay description	Inhibition of EGFR in presence of 2 uM ATP	Binding constant for EGFR(L858R) kinase domain
Assay format	Cell free	Biochemical
Assay type	Functional	Binding
Assay sub type	Enzyme activity	Binding reversible
Detection technology	Spectrophotometry	qPCR
Inhibitor type	Competitive inhibitor	Inverse agonist
End point action	Inhibition	Inhibition
Reference	Bioorg. Med. Chem. Lett., (2006) 16:10	Nat. Biotechnology, (2011) 29:11

Figure S3. (A) Classification of the compound targets with μBAO annotation in the current DTC database based on NCBI taxonomic classification. (B) Classification of the compounds with μBAO annotation in the current DTC database based on their ATC level 2 information on pharmacological action. (C) Bioactivity end-points for the compound-target pairs with μBAO annotation present in the current DTC database. Related to Figure 2.

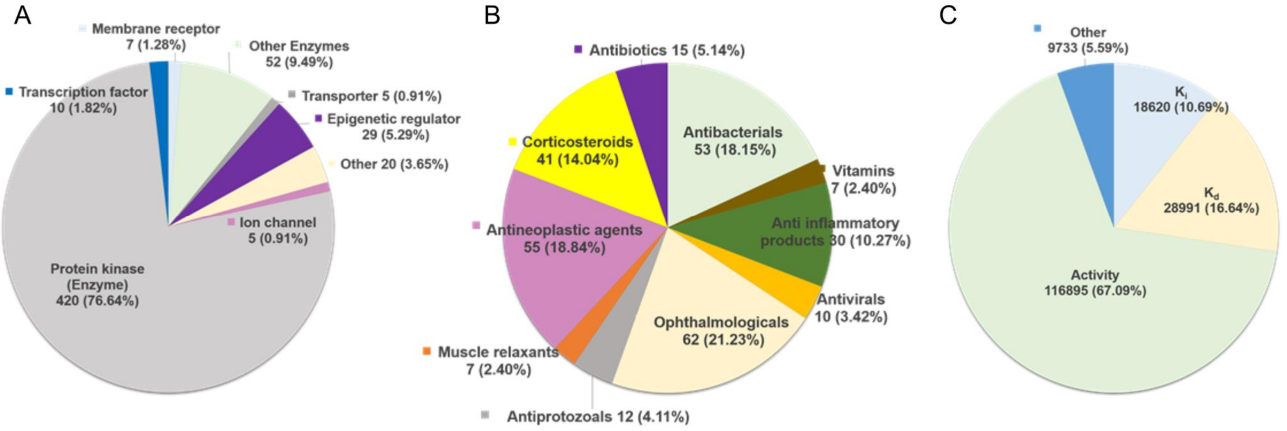

Figure S4. The current DTC µBAO assay annotation statistics in terms of (A) assay types, (B) assay subtypes, (C) assay formats, (D) mode of action, (E) detection technologies, and (F) inhibitor types. These statistics are based on the µBAO annotated DTC bioactivity data points only. Related to Figure 2.

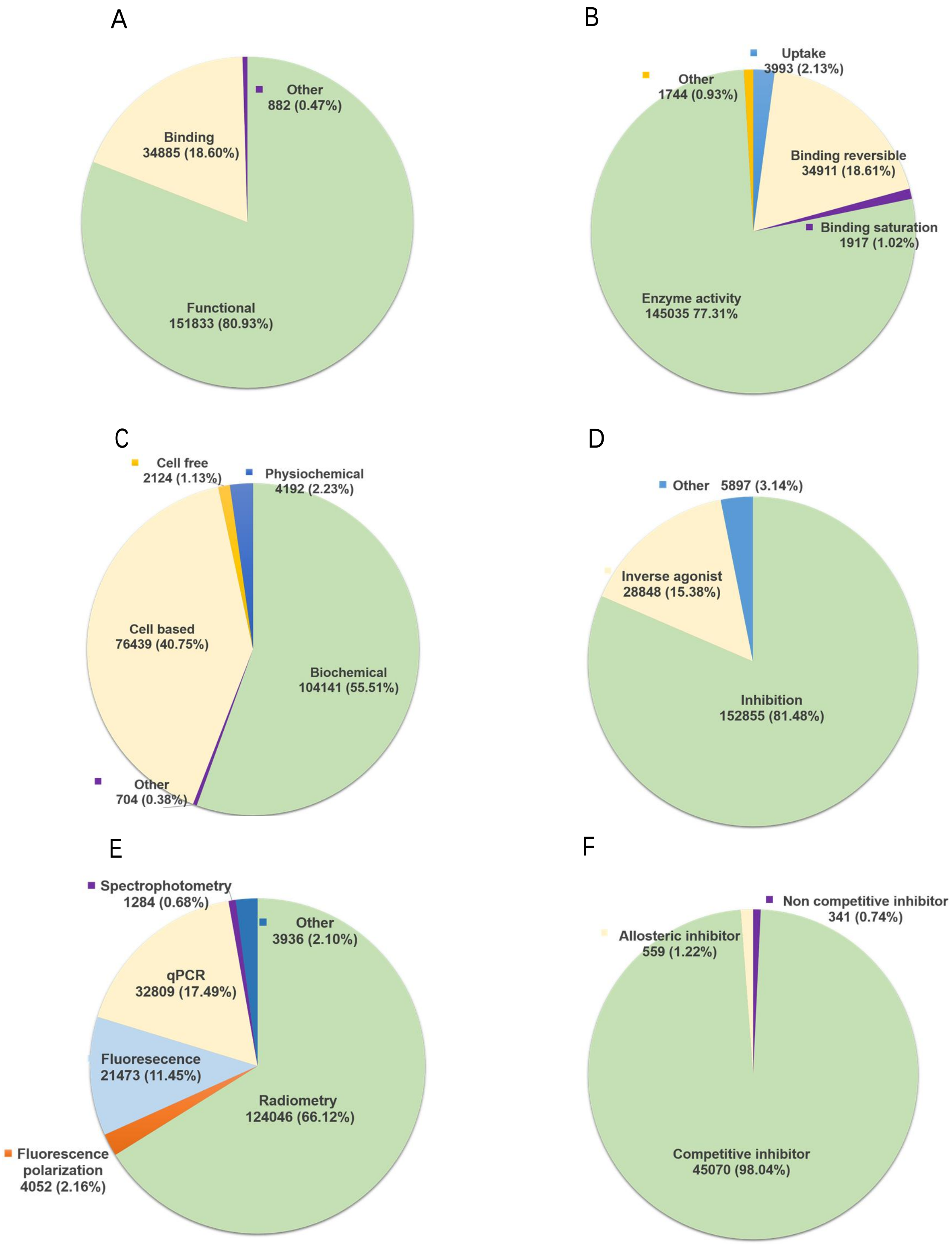

Figure S5. The number of distinct bioactivities (left y-axis) for the top-30 compounds with most bioactivity data annotated in the current DTC release. The colours indicate the four most popular bioactivity end-points (K_d, K_i, IC_{50} or activity). The line-chart shows the number of potent unique targets for each compound (right y-axis). Related to STAR Methods.

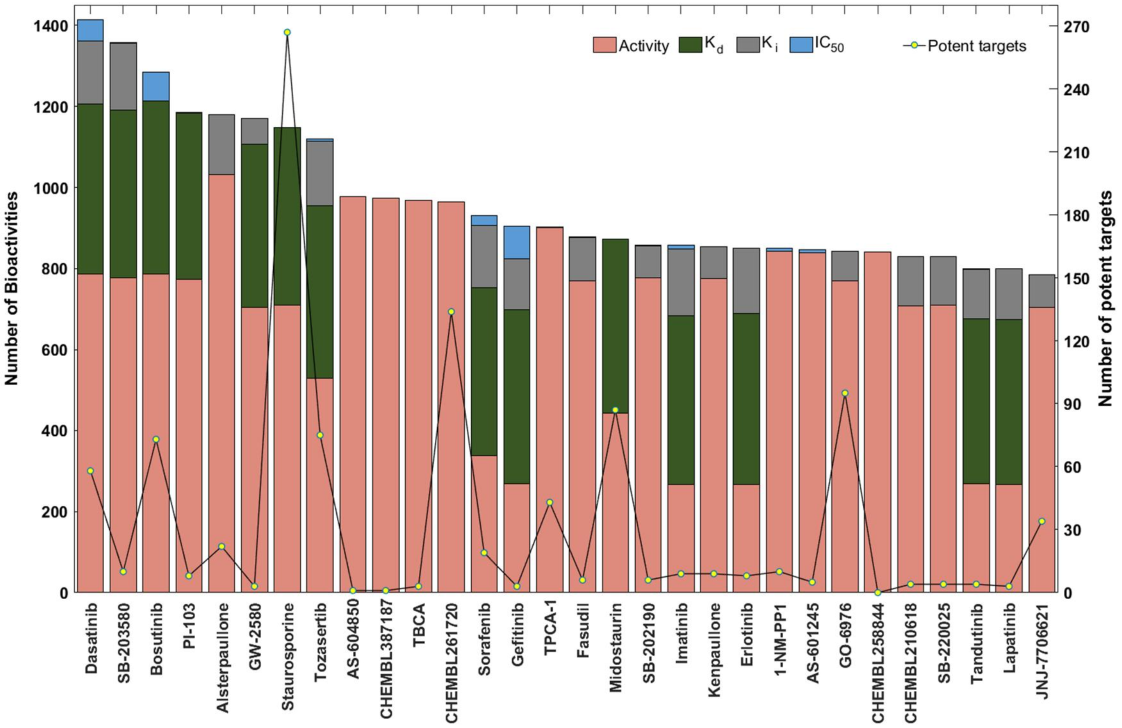

Figure S6. (A) The number of unique active compounds (y-axis) for the top-30 wildtype targets with most annotated bioactivity data (x-axis), where the potent compounds are defined as described in the STAR Methods. (B) The number of unique active compounds (y-axis) for the top-10 disease-related mutated targets with most annotated bioactivity data in the current DTC database (x-axis). The trace plot shows the number of unique mutations in each of the selected targets, and the number of active compounds is summed up over these variants. Related to STAR Methods.

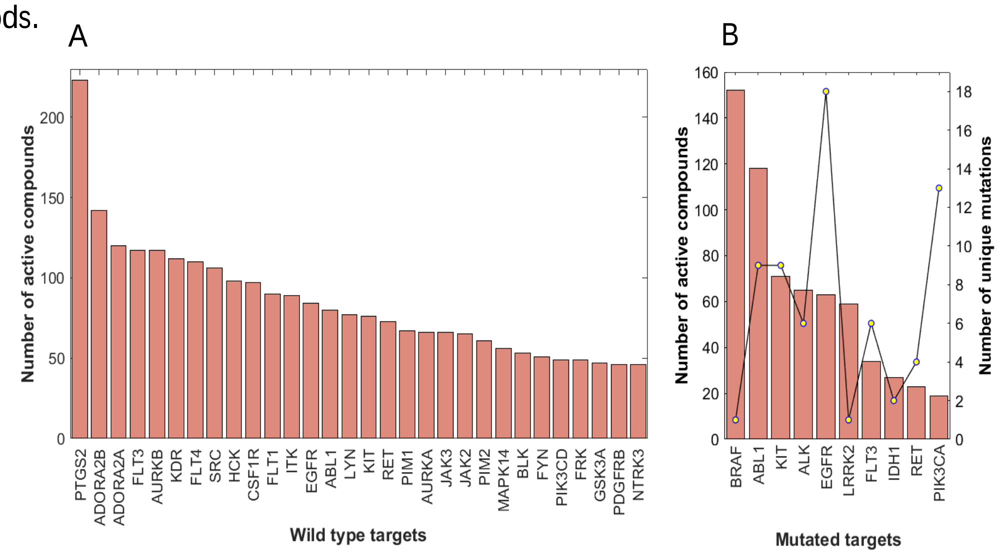

Supplement: Document S1. Figures S1–S6 [file mmc1.pdf]
