# Supplementary material for: Drug Target Commons: A Community Effort to Build a Consensus Knowledge Base for Drug-Target Interactions
Source: Cell Chem Biol. 2018 Feb 15;25(2):224–229.e2. doi: 10.1016/j.chembiol.2017.11.009 (PMC5814751; doi:10.1016/j.chembiol.2017.11.009)
Supplement: Data S1. User Manual for the DTC Web Interface, Related to STAR Methods [file mmc5.zip › Data S1.pdf]

Drug Target Commons

User manual

May, 2017

Table of Contents

Introduction 2

Browser Requirements 2

User Information / Login 2

Home page 3

Search bioactivity data 4

Bulk import for new bioactivity data 8

- Download and edit bioactivity data template 8
- Upload the edited Excel file. 9

Submissions page 10

User feedback 10

- Feedback / Comments form 11

Glossary 11

Downloads 11

Introduction

Drug Target Commons (DTC) is a community-based crowdsourcing effort to improve the consensus on drug-target interactions. The DTC platform contains both a database that stores compound-target bioactivity profiles and bioassay annotations (μBAO), as well as a graphical user interface (GUI) that enables users to upload, edit, curate, annotate and download high-quality, comprehensive bioactivity data.

The DTC database contains two types of bioactivity data, **unannotated and annotated for bioassay ontology**. The DTC GUI allows users to search bioactivity data using compound, target or publication identifiers (IDs). Users may also submit suggestions to edit or add bioactivity data, export, as well as take part in the μBAO bioassay annotation process.

Browser Requirements

DTC has been checked for compatibility with standard modern browsers (Mozilla Firefox, Google chrome, Safari and Internet Explorer).

User Information / Login

DTC data has open access but user information (name and email)/login is required if the user is submitting annotations in order to avoid spam. To login, user can use google account or create a DTC user login.

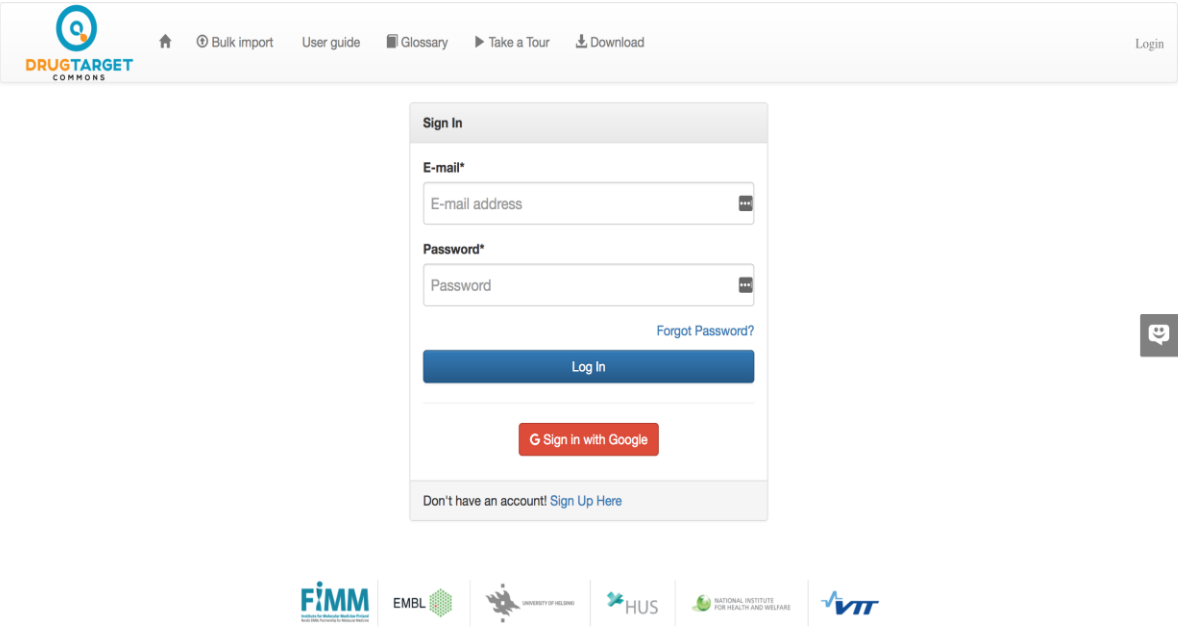

Home page

From the DTC home page, a user can search both unannotated and annotated bioactivity data and view statistics of the database.

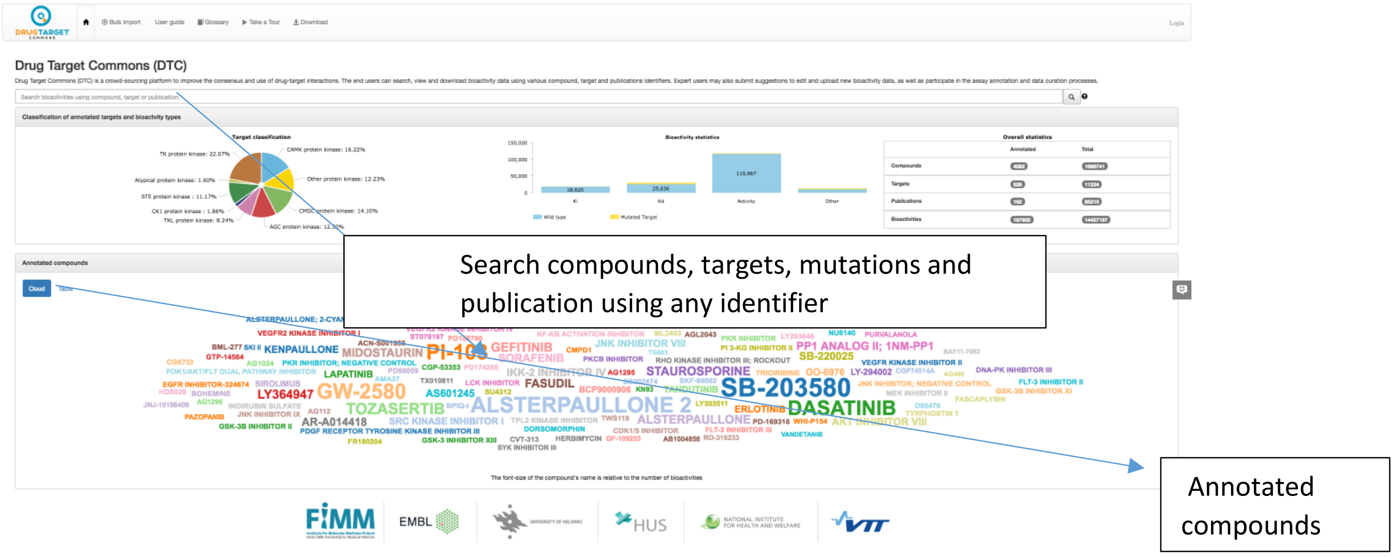

Search bioactivity data

The user can search bioactivity data (annotated and unannotated).
Search keywords are matched to (A) compound, (B) target (C) Pubmed ID and (D) Mutations

(A) **Compound search** can be performed by compound name or synonyms, standard international identifier InChIKey or several other compound IDs (ChEMBL, Bindingdb, Molport, SureChEMBL, Actor, nmrshiftdb2, PubChem_tpharma, kegg_ligand).
- Partial and exact searches are supported for compound name or synonyms (ChEBI, HMdb, fdasrs, Selleck, LINCS, Drugbank, NIC_ncc, PharmGKB, ibm, nikkaji, PubChem, emolecules, zinc, recon, mcule, pubchem_dotf, gtopdb, pdb, pubchem_tpharma, kegg_ligand).
- InChIKey or other IDs must have an exact match.

After entering compound name or ID, the user should click on the search button (or press enter key) to search the present database.

(B) **Target search** can be performed by target name, gene name or protein ID (UniProtKB-AC, UniProtKB-ID, EMBL-CDS, Ensembl, PIR, PubMed, NCBI-taxon, Ensembl_TRS, MIM, UniGene, UniRef50, RefSeq, GO, PDB, UniParc, Additional PubMed, EMBL, GI, GeneID, Chembl, UniRef100, UniRef90, Ensembl_PRO). Other specifications are similar to the compound search. There is the same same filter on target types.

(C) **Publication search** can be done using PubMed ID. If the PubMed ID information is not included in the current database, the user may manually enter the required fields. If the PubMed ID information exists in the system, then the information about compound, targets, bioactivities, assays and publication will be retrieved automatically. The user may filter out irrelevant compounds or targets from the search results.

(D) **Mutation**

E.g. ABL1(T315I)

Step-by-step example of how to search and edit unannotated bioactivity data

Let’s take an example, where the user wants to search and edit the bioactivities related to the key word **“ABL”** (search is case insensitive)

IMPORTANT NOTE: User has to login to use this feature.

Step 1: Enter **“ABL”** in the search box and click search icon (or press enter)

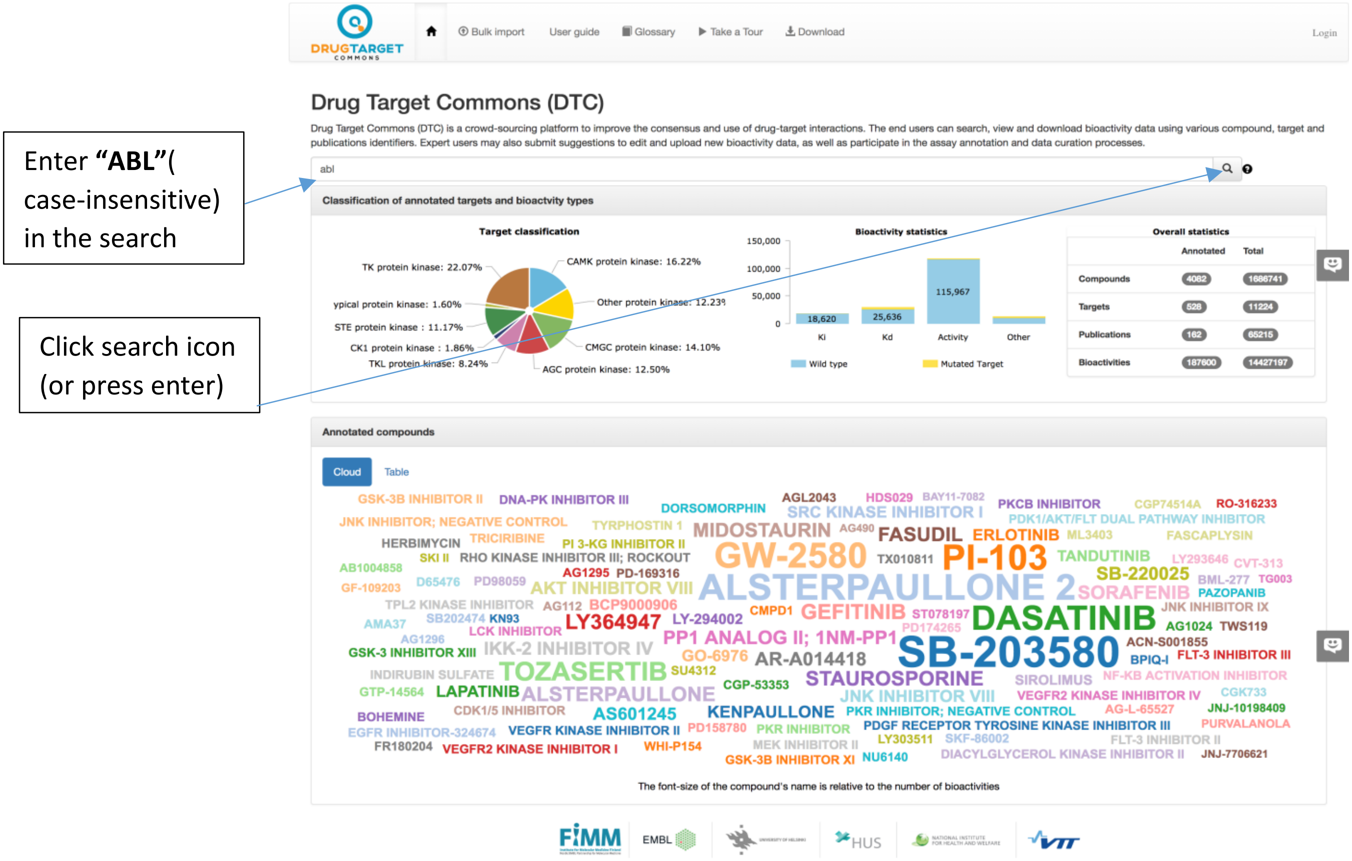

Step 2: The resulting list of compounds and targets are shown with the keyword **"ABL"** (1 entries below). Click **Detail** for the compound **"Tyrosine-protein kinase ABL,"** for example, to view the associated bioactivities.

Click on any column to sort .

Click on the filter icon on a specific column to perform filtering.

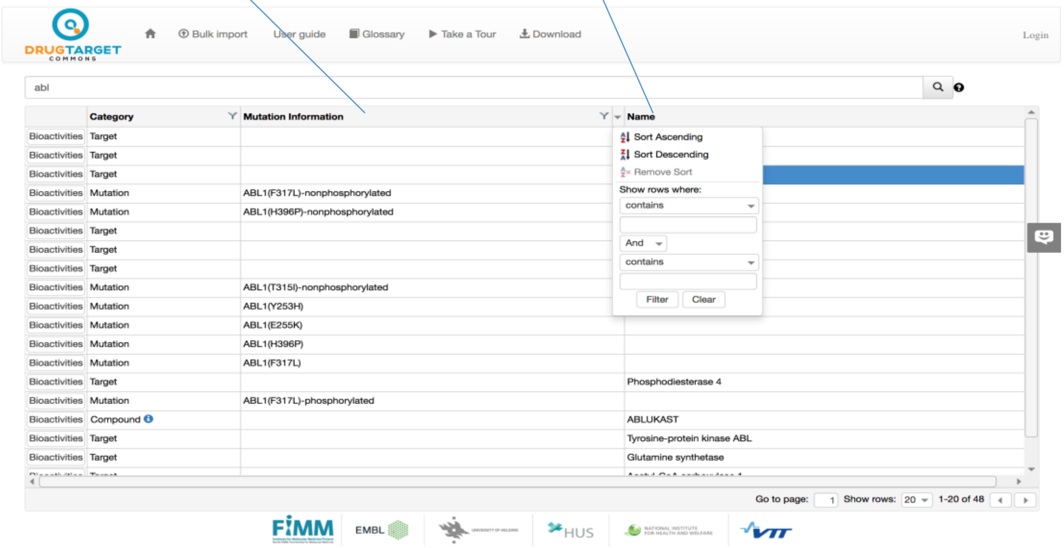

The list of bioactivities for the compound "**Tyrosine-protein kinase ABL"** is then shown**.**

Click on any column to sort .

Click on the filter icon on a specific column to perform filtering.

Status: annotated or unannotated bioactivities

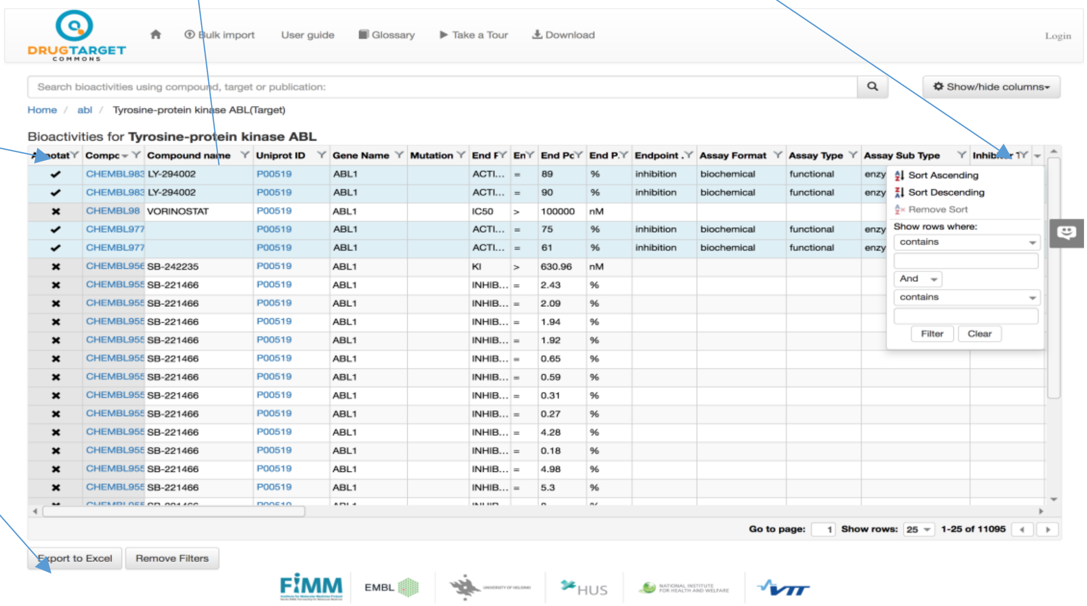

You can export the data into an Excel sheet and edit it in offline mode.

Step 3: Once the user has the desired bioactivities, there are two ways to edit the annotations.

a) Edit online

Click on any column to sort.

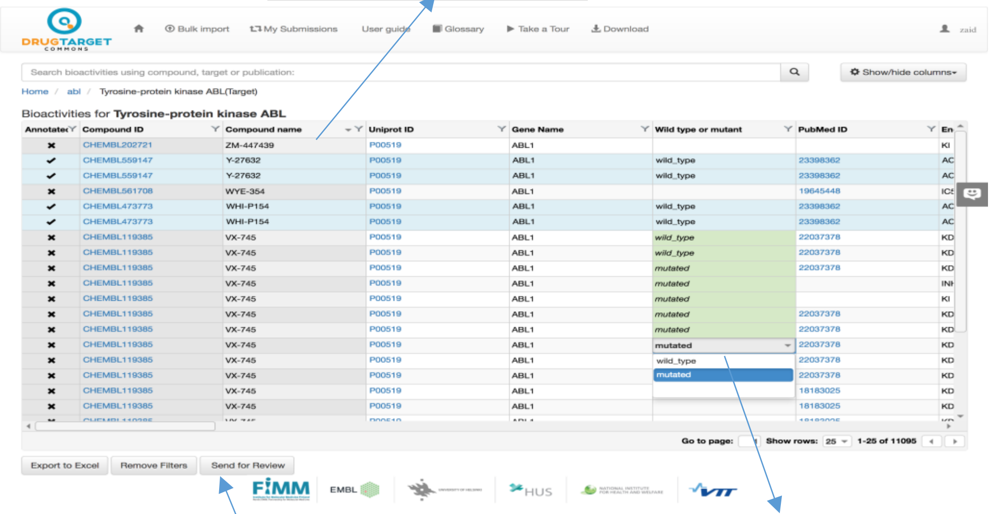

Step 2: Click the **Send for Review** button to submit annotations to the DTC administrators.

Step 1: Edit by clicking on any column which turns green once edited.

b) **Edit offline** by downloading the data into an Excel sheet (useful with a larger data set, where one can utilize Excel features to do more complex editing tasks), and then uploading the data back to DTC using the bulk import feature.

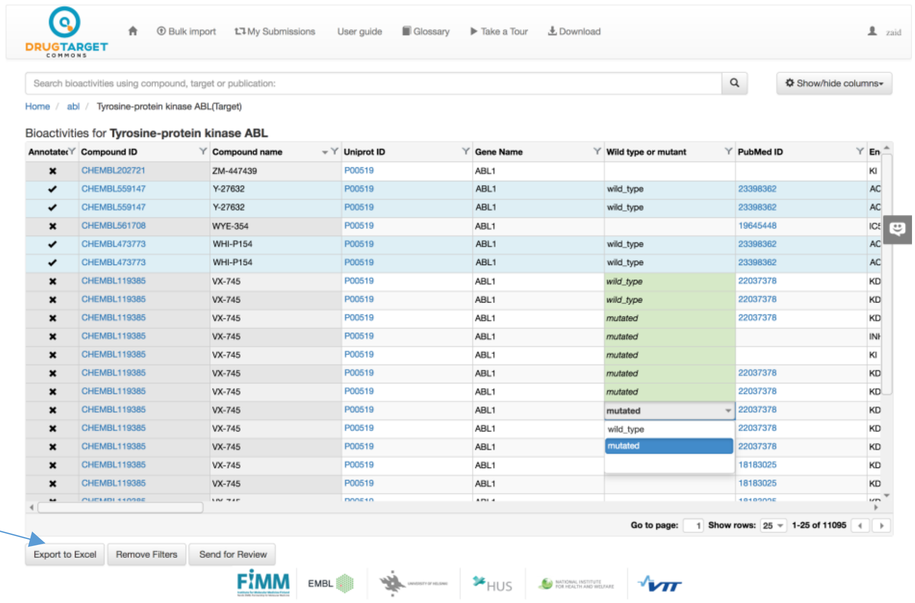

The user can export the data into an Excel sheet and edit it offline in Excel.

Bulk import for new bioactivity data

Bulk import of bioactivity data occurs in two stages: download and edit the bioactivity data template file, and upload of the new file.

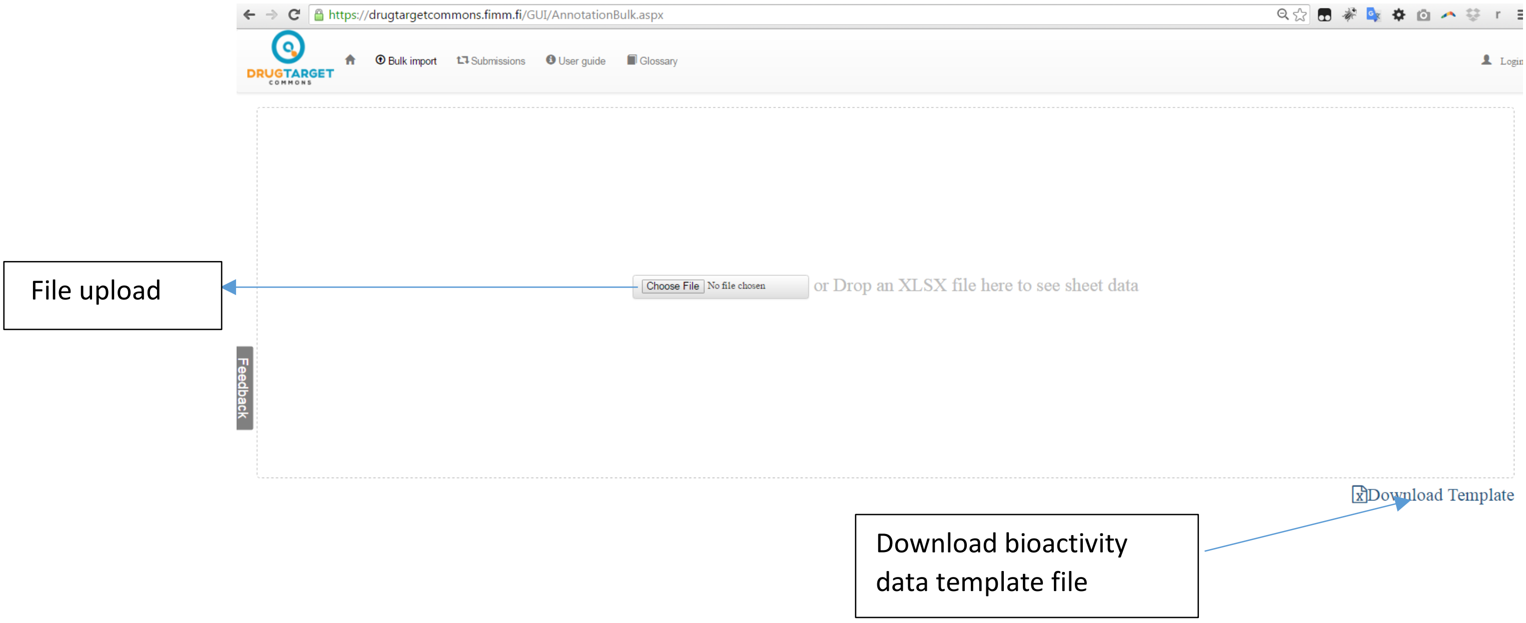

Download and edit bioactivity data template

Step 1: Click on the **Download Template** link and download the Excel file.
Step 2: Fill in the columns in the file with required bioactivity data and assay annotations.

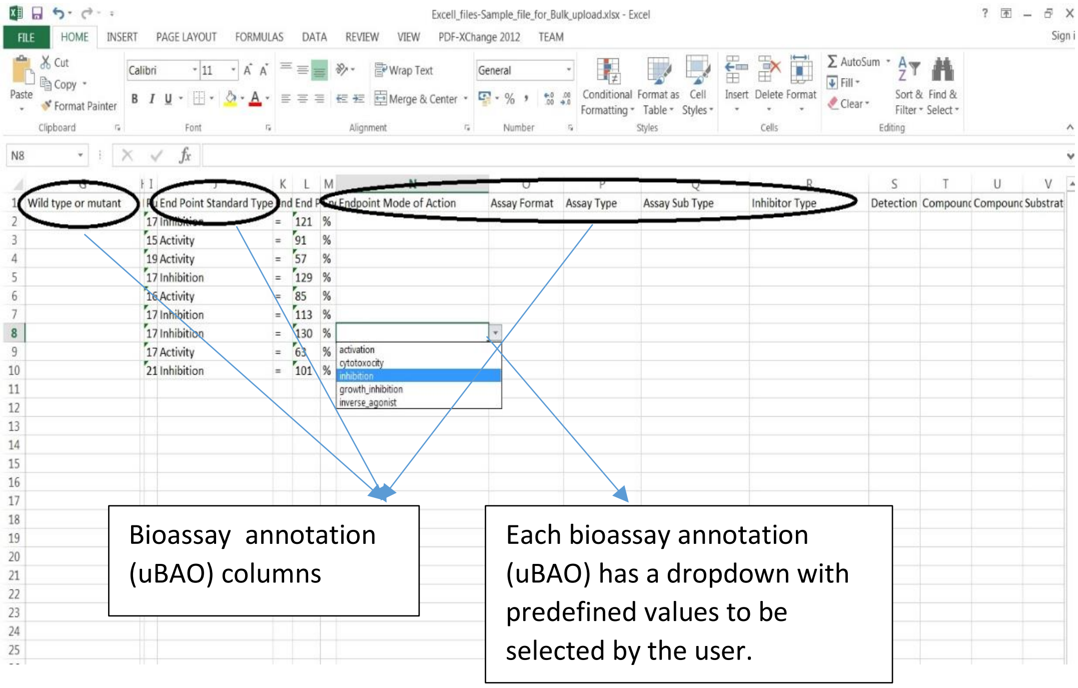

IMPORTANT NOTE: User should strictly follow the Excel file format and should not change the column headers, otherwise the data will not be uploaded successfully.

Upload the edited Excel file.

Step 1: Click to upload the new file or drag and drop the Excel file in the dotted box

Step 2: The uploaded data will be visible as in the below example

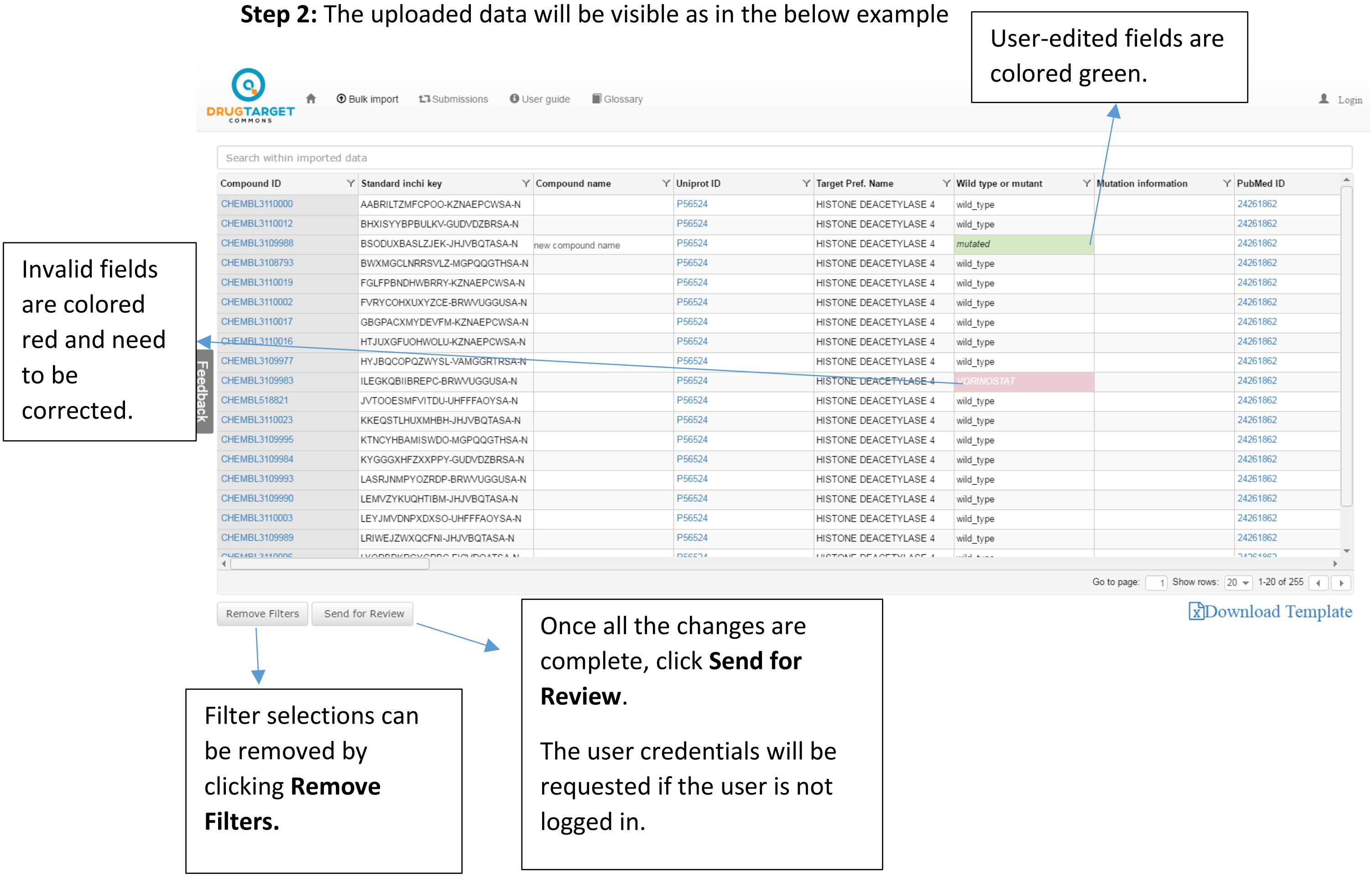

Submissions page

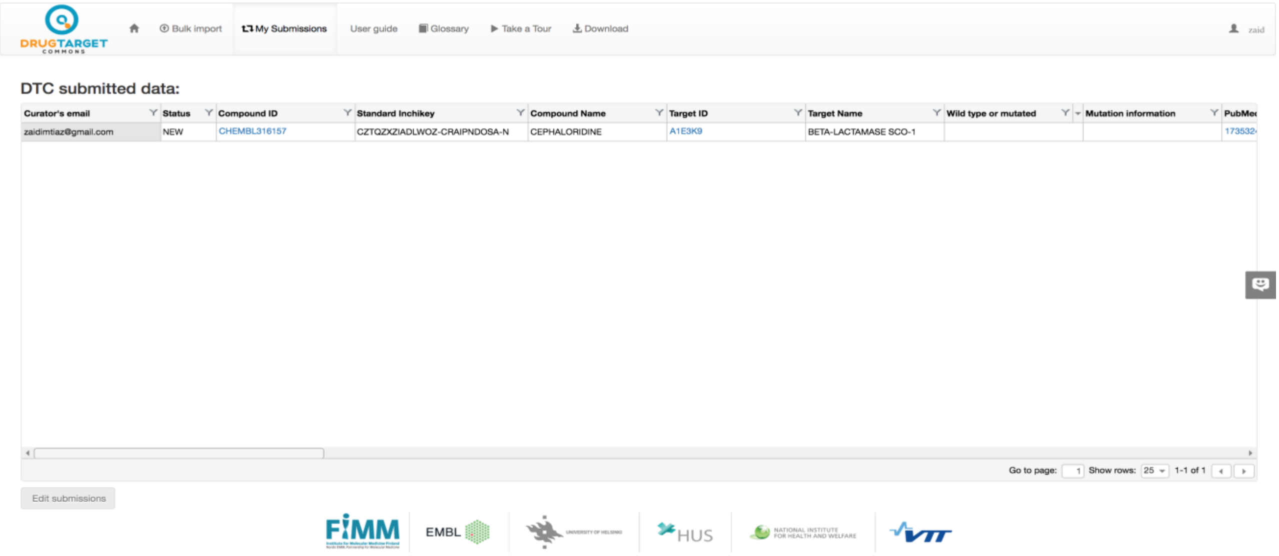

All of the submitted bioactivity annotations by a user can be viewed in the submission page. Submissions will be then reviewed and approved or rejected by the DTC administrators.

User feedback

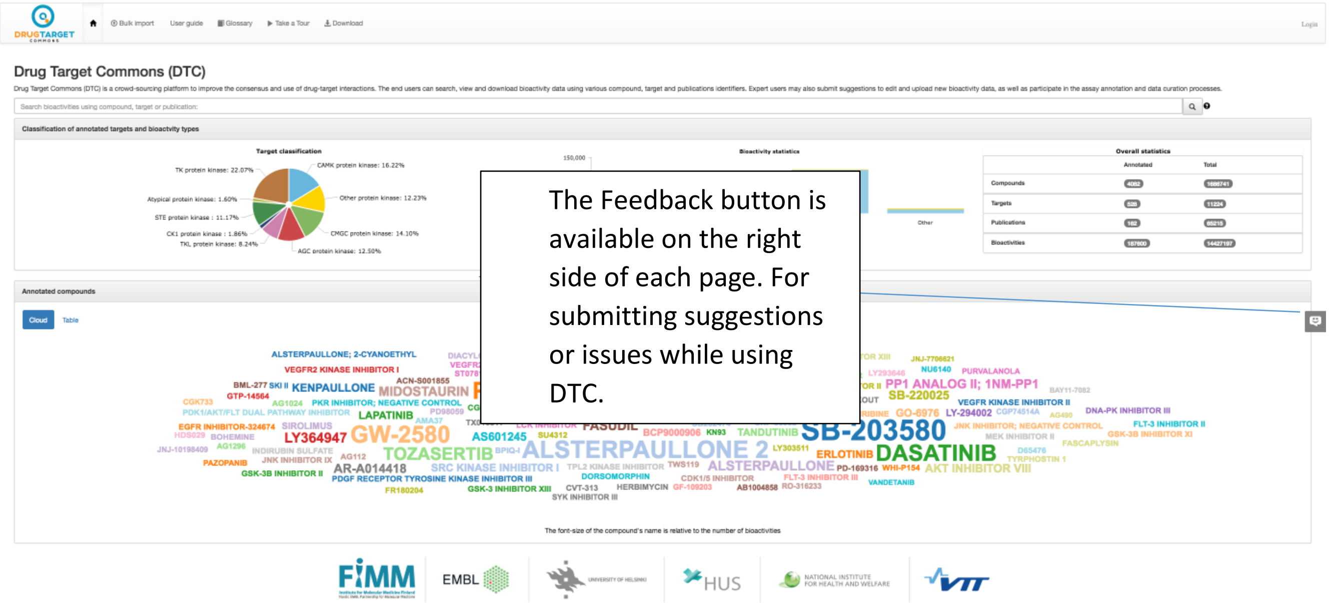

Feedback / Comments form

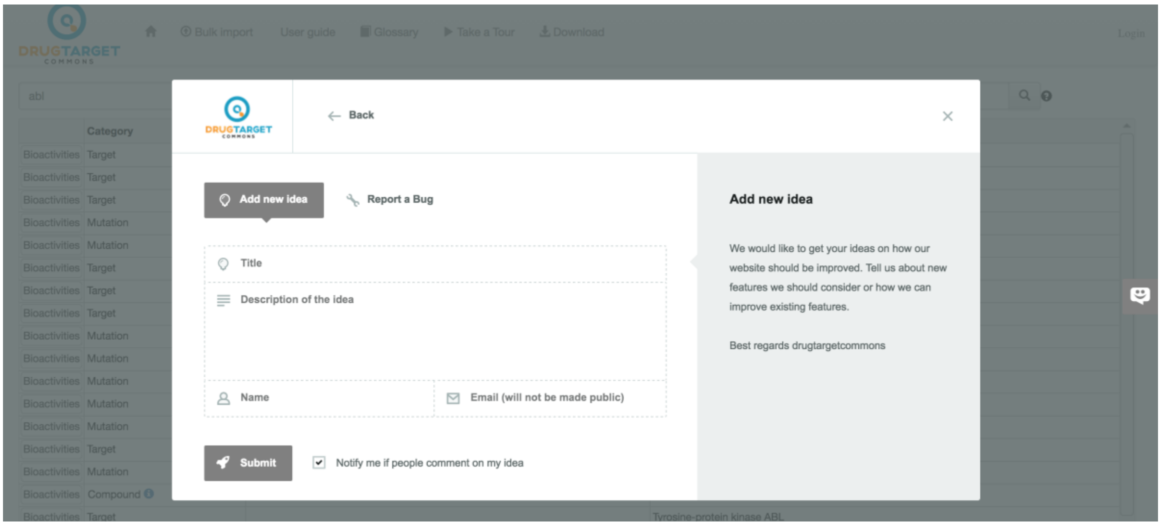

Glossary

A brief explanation of the bioassay annotation terms.

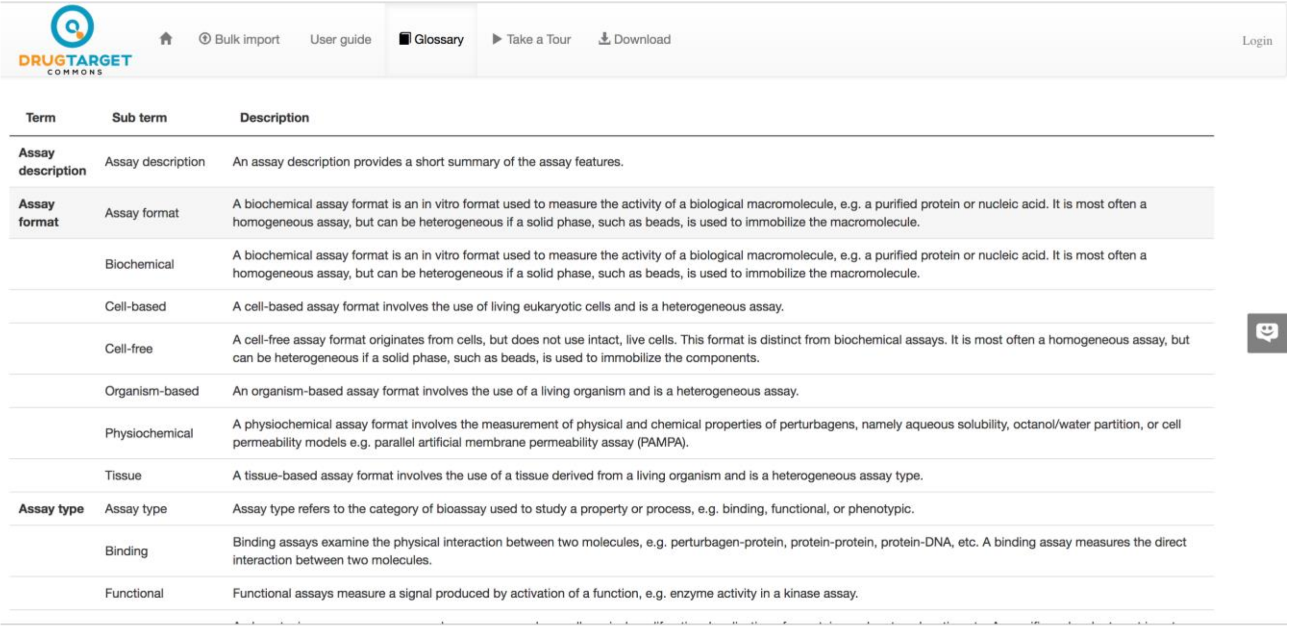

Term	Sub term	Description
**Assay description**	Assay description	An assay description provides a short summary of the assay features.
**Assay format**	Assay format	A biochemical assay format is an in vitro format used to measure the activity of a biological macromolecule, e.g. a purified protein or nucleic acid. It is most often a homogeneous assay, but can be heterogeneous if a solid phase, such as beads, is used to immobilize the macromolecule.
	Biochemical	A biochemical assay format is an in vitro format used to measure the activity of a biological macromolecule, e.g. a purified protein or nucleic acid. It is most often a homogeneous assay, but can be heterogeneous if a solid phase, such as beads, is used to immobilize the macromolecule.
	Cell-based	A cell-based assay format involves the use of living eukaryotic cells and is a heterogeneous assay.
	Cell-free	A cell-free assay format originates from cells, but does not use intact, live cells. This format is distinct from biochemical assays. It is most often a homogeneous assay, but can be heterogeneous if a solid phase, such as beads, is used to immobilize the components.
	Organism-based	An organism-based assay format involves the use of a living organism and is a heterogeneous assay.
	Physiochemical	A physiochemical assay format involves the measurement of physical and chemical properties of perturbagens, namely aqueous solubility, octanol/water partition, or cell permeability models e.g. parallel artificial membrane permeability assay (PAMPA).
	Tissue	A tissue-based assay format involves the use of a tissue derived from a living organism and is a heterogeneous assay type.
**Assay type**	Assay type	Assay type refers to the category of bioassay used to study a property or process, e.g. binding, functional, or phenotypic.
	Binding	Binding assays examine the physical interaction between two molecules, e.g. perturbagen-protein, protein-protein, protein-DNA, etc. A binding assay measures the direct interaction between two molecules.
	Functional	Functional assays measure a signal produced by activation of a function, e.g. enzyme activity in a kinase assay.

Downloads

Download all data in tab-delimited text file.
