# Supplementary material for: Drug Target Commons: A Community Effort to Build a Consensus Knowledge Base for Drug-Target Interactions
Source: Cell Chem Biol. 2018 Feb 15;25(2):224–229.e2. doi: 10.1016/j.chembiol.2017.11.009 (PMC5814751; doi:10.1016/j.chembiol.2017.11.009)
Supplement: Data S2. Glossary of μBAO Annotation Terms, Related to STAR Methods [file mmc6.zip › Data S2 .pdf]

DTC Glossary

Term	Sub term	Description
**Assay description**		An assay description provides a short summary of the assay features.
**Assay format**		An assay format is a conceptualization of assays based on the biological and/or chemical features of the experimental system. For example, assay formats include 1) biochemical, assays with purified protein, 2) cell-based, assays in whole cells, 3) cell-free, assays in cell derivatives, 4) organism-based, assays performed in an organism, 5) physiochemical, assays which measure physical or chemical properties, and 6) tissue-based, assays using tissue derived from a living organism.
	Biochemical	A biochemical assay format is an *in vitro* format used to measure the activity of a biological macromolecule, e.g. a purified protein or nucleic acid. It is most often a homogeneous assay, but can be heterogeneous if a solid phase, such as beads, is used to immobilize the macromolecule.
	Cell-based	A cell-based assay format involves the use of living eukaryotic cells and is a heterogeneous assay.
	Cell-free	A cell-free assay format originates from cells, but does not use intact, live cells. This format is distinct from biochemical assays. It is most often a homogeneous assay, but can be heterogeneous if a solid phase, such as beads, is used to immobilize the components.

	Organism-based	An organism-based assay format involves the use of a living organism and is a heterogeneous assay.
	Physiochemical	A physiochemical assay format involves the measurement of physical and chemical properties of perturbagens, namely aqueous solubility, octanol/water partition, or cell permeability models e.g. parallel artificial membrane permeability assay (PAMPA).
	Tissue	A tissue-based assay format involves the use of a tissue derived from a living organism and is a heterogeneous assay type.
**Assay type**		Assay type refers to the category of bioassay used to study a property or process, e.g. binding, functional, or phenotypic.
	Binding	Binding assays examine the physical interaction between two molecules, e.g. perturbagen-protein, protein-protein, protein-DNA, etc. A binding assay measures the direct interaction between two molecules.
	Functional	Functional assays measure a signal produced by activation of a function, e.g. enzyme activity in a kinase assay.
	Phenotypic	A phenotypic assay measure a complex response such as cell survival, proliferation, localization of a protein, nuclear translocation etc. A specific molecular target is not known or assumed.
**Assay subtype**	Binding, reversible	In a reversible binding assay, the interaction of a ligand with a receptor is dissociated by

		altering the binding conditions, e.g. buffer, salt concentration, etc.
	Binding, irreversible	In an irreversible binding assay, the interaction of a ligand with a receptor is permanent and cannot be dissociated by changing the binding conditions.
	Binding, saturation	A saturation binding assay measures specific radiolabelled ligand binding at equilibrium at various concentrations.
	Enzyme activity	An enzyme activity assay is used to measure the effect of a perturbagen on enzyme activity, measured either directly or as coupled to a secondary molecule.
	Process	A process refers to a recognized chemical reaction or molecular function with a defined beginning and end. Examples include RNA splicing, proteolysis, cell death, neurite outgrowth, etc.
	Reporter gene	A reporter gene assay measures gene expression from an artificial gene construct containing the coding sequence for a protein whose expression can be tracked or reported. In the DNA construct, the coding sequence of a reporter gene is inserted under the control of a promoter or regulatory element of interest. Reporter genes include luciferase, beta galactosidase, beta lactamase, chloramphenicol acetyl transferase, or a fluorescent protein.
	Signaling	A signaling assay measures one of the series of events that are downstream from a chemical signal at the cell surface, e.g. formation of cyclic AMP, transcription factor activation, cytokine secretion, etc., which results in a

		specific cellular activity, such as cell division, cell migration, etc.
	Uptake	An uptake assay refers to the measurement of a process where a substance is taken up by a cell, most commonly by receptor-mediated endocytosis.
	Viability	A viability assay measures the cellular state of living or dying by using an indicator of life or death. Cell life indicators include ATP content, cell number, dehydrogenase activity, DNA content, esterase activity, mitochondrial membrane potential, membrane integrity, and protease activity. Cell death indicators include caspase activity, chromatin condensation, and phospholipid redistribution
**Detection technology**		A detection technology is the physical method or technique readout used to measure an effect caused by a perturbagen in an assay environment.
	Fluorescence	Fluorescence detection methods use the principles of fluorescence, whereby incident light excites a fluorophore that then emits light at lower energy and higher wavelength, typically in the visible portion of the UV-Visible spectrum.
	Fluorescence polarization (FP)	Fluorescence polarization (FP) measurements are based on the assessment of size-dependent rotational motions of species and used to measure binding interactions.
	AlphaScreen	Amplified Luminescent Proximity Homogeneous Assay Screen is a subtype of fluorescence detection technologies.

	Time Resolved Fluorescence (TRF)	Time Resolved Fluorescence (TRF) is a subtype of fluorescence detection technologies. One commercial name is DELFIA.
	Time -Resolved Fluorescence Energy Transfer (TR-FRET)	Time-Resolved Fluorescence Energy Transfer (TR-FRET) is a subtype of fluorescence detection technologies. Commercial names are LANCE and HTRF.
	Label-free technology	Label-free detection technologies measure binding interactions and cell-based reactions in the absence of conventional labels, e.g., fluorescent probes. Advantages include the ability to measure a) functional activity without modifying the binding partners with labels, b) binding interactions independent of functional activity, and c) cell-based assays without the need to engineer cell-lines to over-express given targets, such as GPCRs.
	Luminescence	Luminescence detection technologies make use of light emission that occurs from an electronically excited state reached by a physical, mechanical, or chemical mechanism.
	Microscopy	Microscopic detection technologies use microscopes to see objects that cannot be seen with an unaided eye.
	Quantitative PCR (qPCR)	Quantitative PCR, sometimes referred to as RT-PCR, detection technologies use DNA labeled protein in a binding assay and are detected by quantifying the amount of DNA by PCR. A commercial assay called KINOMEscan was specifically developed for screening purposes.

	Radiometry	Radiometry detection technologies use radioactive tracers. Examples of assays that use radiometry are filter assays and Scintillation Proximity Assay (SPA).
	Spectrophotometry	Spectrophotometry detection technologies measure the amount of light that a sample absorbs. A spectrophotometer operates by passing a beam of light through a sample and measuring the intensity of light reaching a detector.
	Thermal shift	Thermal shift detection technologies detect temperature shifts using a fluorescent dye that is sensitive to a protein environment. Upon heating, a protein unfolds and loses native conformation. Binding of a small molecule can often stabilize the protein conformation, resulting in a higher unfolding temperature.
**Endpoint mode of action**		An assay endpoint mode of action refers to the effect of the perturbagen on the assay target, e.g. inhibition, activation, cytotoxicity, etc.
	Activation	An activation endpoint action is the initiation, enhancement, or increase of a biological action, such as increasing the rate of an enzymatic reaction or positively regulating gene expression through an increased rate of transcription.
	Cytotoxicity	A cytotoxicity endpoint action is a toxic or cell death-inducing property of a perturbagen. Known cytotoxic agents mediate cell death commonly by inducing necrosis, apoptosis, or autophagy. Cytotoxicity can be detected by measuring various aspects of the death process, e.g. membrane permeability, ATP concentration, DNA fragmentation, etc.

	Growth inhibition	A growth inhibition endpoint action refers to growth reduction of a predefined stimulus. The unit of measure is always percent, when normalized to the dynamic range of the assay. Cell viability or proliferation can be measured in a variety of ways, e.g. 1) protein content by sulforhodamine B staining followed by absorbance measurement, 2) mitochondrial dehydrogenase activity by 3-(4,5-dimethylthiazol-2-yl)-2,5-diphenyl tetrasodium bromide, MTT staining, followed by absorbance measurement, 3) expression of proliferation-associated antigens by immunostaining for Ki-67, and 4) ATP content by using CellTiter-Glo reagent (Promega) followed by luminescence measurement.
	Inhibition	An inhibition endpoint action refers to reduction of a predefined stimulus.
	Inverse agonist	An inverse agonist endpoint action refers to a modulator that binds to the same receptor as an agonist, but induces a response opposite to that of an agonist.
**Inhibitor type**		The inhibitor type describes the mode of action of the inhibitor; e.g., many kinase inhibitors are ATP competitors.
	Competitive inhibitor	A competitive inhibitor binds to the active site of the enzyme and prevents substrate from

		binding. A competitive inhibitor can only bind to free enzyme.
	Noncompetitive inhibitor	A noncompetitive inhibitor binds to the enzyme at a site distinct from the active, substrate-binding, site. A noncompetitive inhibitor can bind to free enzyme or an enzyme-substrate complex.
	Allosteric inhibitor	An allosteric inhibitor decreases activity or substrate affinity by binding to a site distinct from the active site, an allosteric site. This interaction is characterized by a conformational change in the target that is required for inhibition.
**Substrate type**		Substrate is needed in order for the enzyme to be able to produce a product, most likely measurable when considering bioassays. Some common substrates are: ATP for kinases, methyl group donors for methylases, acetyl group donors for acetylases, GTP for GTPases, NADH or NADPH for dehydrogenases, or numerous other metabolites, small molecules, peptides, proteins, etc.
**Assay cell line**		The cell line used for the assay (cell line IDs or cell line ontology)
